# Anthracene appended pyridinium amide–urea conjugate in selective fluorometric sensing of L-*N-*acetylvaline salt

**DOI:** 10.3762/bjoc.6.139

**Published:** 2010-12-21

**Authors:** Kumaresh Ghosh, Tanmay Sarkar, Asoke P Chattopadhyay

**Affiliations:** 1 Department of Chemistry, University of Kalyani, Kalyani-741235, India, Fax +91-33-25828282

**Keywords:** *N*-acetyl-L-valine salt, DFT calculation, emission decay, fluorometric detection, pyridinium amide–urea conjugate

## Abstract

A new anthracene labeled pyridinium amide–urea conjugate **1** has been designed and synthesized. The receptor shows a different fluorometric response with L-*N*-acetylvaline and L-*N-*acetylalanine salts in CH_3_CN in contrast to the other salts of L-*N-*acetyl α-amino acids and (*S*)-α-hydroxy acids studied. Upon complexation of the tetrabutylammonium salt of L-*N*-acetylvaline, the emission of **1** increases accompanied by the formation of a new band at higher wavelength and this characteristic change distinguishes it from other anionic substrates studied. The binding interaction has been studied by ^1^H NMR, fluorescence and UV titration experiments.

## Introduction

The design and synthesis of artificial receptors capable of recognizing α-hydroxy and *N-*acetyl-α-amino acid carboxylates (i.e., salts of α-amino acids) is an active area of interest in supramolecular chemistry due to the biological significance and practical importance of α-amino and α-hydroxy acids [1–7]. While α-hydroxycarboxylic acids are useful synthons for many organic natural products and drug molecules, α-amino carboxylic acids are important as they are the building blocks of proteins, enzymes etc., which govern numerous biochemical processes. In this context, detection and sensing of these molecules by fluorescence spectroscopy offers a variety of advantages such as different detection modes (fluorescence quenching, enhancement and life time), high sensitivity, low instrumentation cost, time efficiency, and the possibility of performing real time analysis. However, the development of receptors for these molecules is slow due to their insolubility in organic solvents, especially amino acids. For strong complexation of zwitterionic amino acids, synthetic receptors should possess complementary binding sites for both ammonium and carboxylate functionalities. Examples in this domain are known in the literature [8–15]. To overcome the solubility problem, α-amino acids are sometimes converted into their *N-*acetyl derivatives which makes their recognition easier in organic solvents [16–19], as these are non-competitive solvents and guests exhibit minimal solubility. Hamilton et al. introduced simple pyridine amide-based receptors for the effective recognition of *N*-acetyl-α-amino acids by multiple hydrogen bonding interactions [20]. During our work on selective recognition of different anionic species including carboxylates [21–22], we reported receptors of various structures with different binding sites. The pyridinium motif, which was first used by Jeong et al. for carboxylate binding [23], was one of the binding sites in our designed receptors [24–26]. The pyridinium motif is unique due to its contribution to the charge–charge interaction and formation of unconventional hydrogen bonds with the anionic guests [27]. In order to explore this binding site for a wide range of substrates, especially for amino acid derivatives, we report here the design and synthesis of a new fluororeceptor **1** where anthracene is attached to the binding site through a covalent CH_2_ spacer to yield a photo induced electron transfer sensory system [28]. The receptor **1** shows effective binding of the tetrabutylammonium salt of L-*N*-acetylvaline by exhibiting significant change in emission.

**Figure 1:**
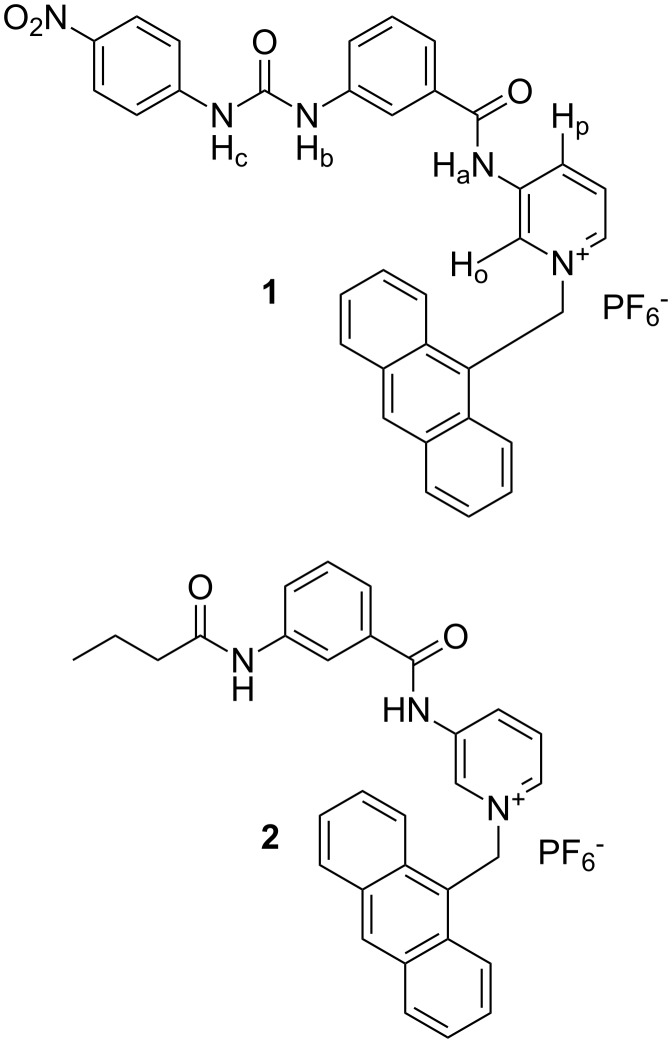
Synthesized compounds **1** and **2**.

Complexation induced formation of an exciplex or charge transfer complex in CH_3_CN is the key feature in the present study for the selective detection of a L-*N-*acetylvaline salt from other anionic guests. To explain the formation of an exciplex or charge transfer complex in **1** upon complexation of L-*N-*acetylvaline salt, compound **2** was synthesized and studied (see Figure 1).

## Results and Discussion

**Scheme 1:**
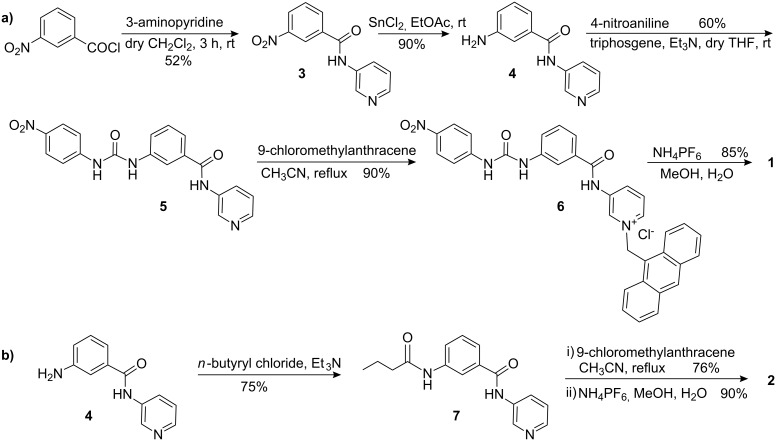
Syntheses of **1** and **2**.

Compounds **1** and **2** were synthesized according to Scheme 1. Initially, 3-nitrobenzoyl chloride was reacted with 3-aminopyridine to give the amide **3**. Reduction of the –NO_2_ group in **3** with SnCl_2_ in EtOAc afforded the amine **4**, which was further reacted with 4-nitrophenyl isocyanate (obtained from 4-nitroaniline by reaction with triphosgene in dry THF) in dry THF to give urea derivative **5**. Subsequent reaction of **5** with 9-chloromethylanthracene under refluxing conditions in dry CH_3_CN gave the chloride salt **6**. Anion exchange of the salt **6** with NH_4_PF_6_ afforded the desired receptor **1** as a white solid. Compound **2** was obtained from the intermediate amine **4** after performing a series of reactions such as amide formation, alkylation on pyridine ring nitrogen followed by anion exchange with NH_4_PF_6_ (Scheme 1b). Compounds **1** and **2** were characterized by ^1^H NMR, ^13^C NMR, FTIR and mass spectrometry.

**Figure 2:**
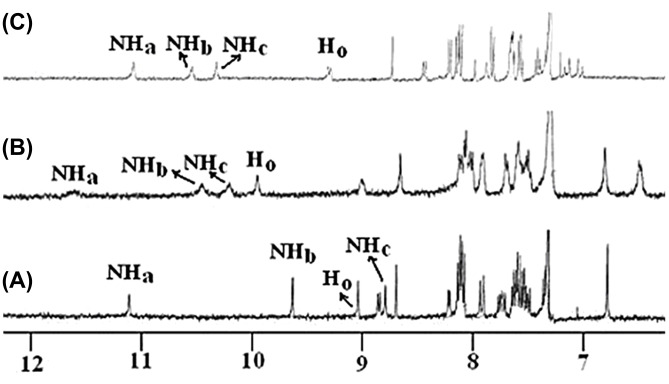
Change in ^1^H NMR of (A) **1** (400 MHz, CDCl_3_ containing 0.4% *d*_6_-DMSO; *c* = 2.80 × 10^−3^ M) and in the presence of equivalent amount of (B) L-*N*-acetylvaline, (C) L-*N*-acetylproline salts.

The solution phase binding interaction of the tetrabutylammonium salts of L-*N*-acetylalanine, L-*N*-acetylvaline, L-*N*-acetylproline and L-*N*-acetylphenylglycine, (*S*)-mandelic and pyruvic acids was investigated by ^1^H NMR, UV–vis and fluorescence techniques. Initially, we recorded the ^1^H NMR of **1** in the presence and the absence of the guests in CDCl_3_ containing 0.4% *d*_6_-DMSO (due to insolubility of **1** in pure CDCl_3_). In the presence of equivalent amounts of all the guests, urea (Δδ_NHb_ = 0.93–1.90, Δδ_NHc_ = 0.87–1.96) and amide protons (Δδ_NHa_ = 0.46–0.79) underwent downfield shifts suggesting their involvement in the binding process. In addition, the pyridinium *ortho* proton (H_o_) in **1** was also downfield shifted (Δδ = 0.83–1.06) which indicated its involvement in the formation of strong hydrogen bonding with the carboxylate moiety of the guests. In contrast, the pyridinium *para* proton (H_p_) showed a small downfield shift (Δδ = 0.02–0.48). This small change in chemical shift of the *para* proton is due to either a change in bond length of the intramolecular hydrogen bond between the amide carbonyl oxygen and *para* hydrogen of pyridinium motif, or involvement in the formation of a hydrogen bond with the guest in solution. On addition of an equivalent amount of tetrabutylammonium salts of acetate and pyruvate to the solution of **1**, precipitation occurred. A similar finding was observed in the presence of an equivalent amount of the alanine salt. Figure 2 shows the change in ^1^H NMR spectrum of **1** in presence of an equivalent amount of tetrabutylammonium salts of L-*N-*acetylvaline, L-*N*-acetylproline and (*S*)-mandelic acid.

**Figure 3:**
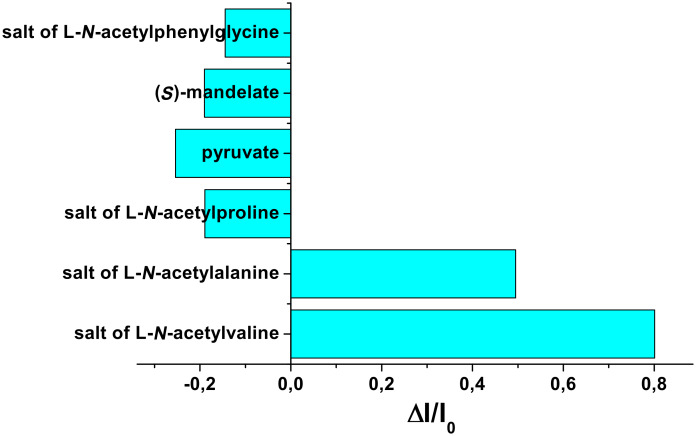
Change in fluorescence ratio of **1** upon addition of one equivalent of anions (*c* = 4.31 × 10^−5^ M) at 492 nm.

Once it was realized that both urea and pyridinium sites of **1** are involved in hydrogen bonding with the guests studied, we recorded the emission spectra of **1** in CH_3_CN. Receptor **1** showed an intense emission at 415 nm when excited at 370 nm in CH_3_CN. Upon gradual addition of the guests to a solution of **1** (*c* = 4.31 × 10^−5^ M) in CH_3_CN, the emission at 415 nm was changed differently. For all guests, except the salts of L-*N*-acetylvaline and L-*N*-acetylalanine, emission of **1** at 415 nm decreased gradually (Supporting Information File 1). However, in the case of the L-*N*-acetylvaline salt, a broad emission at 492 nm with moderate intensity was observed. Figure 3 displays the change in emission of **1** at 492 nm in the presence of one equivalent of each guest in CH_3_CN. The increase in emission of **1** at 492 nm in the presence of tetrabutylammonium salts of L-*N*-acetylvaline and L-*N*-acetylalanine is moderate and distinguishable from the other guests. It is worth noting that the appearance of the new emission at 492 nm is more significant in the presence of L-*N-*acetylvaline salt than with L-*N*-acetylalanine. This is attributed to the formation of a charge transfer complex between the excited state of anthracene and the electron deficient nitrophenyl urea during the interaction process. We believe that this characteristic feature with L-*N*-acetylvaline is due to its structural feature that controls the distance between donor and acceptor.

**Figure 4:**
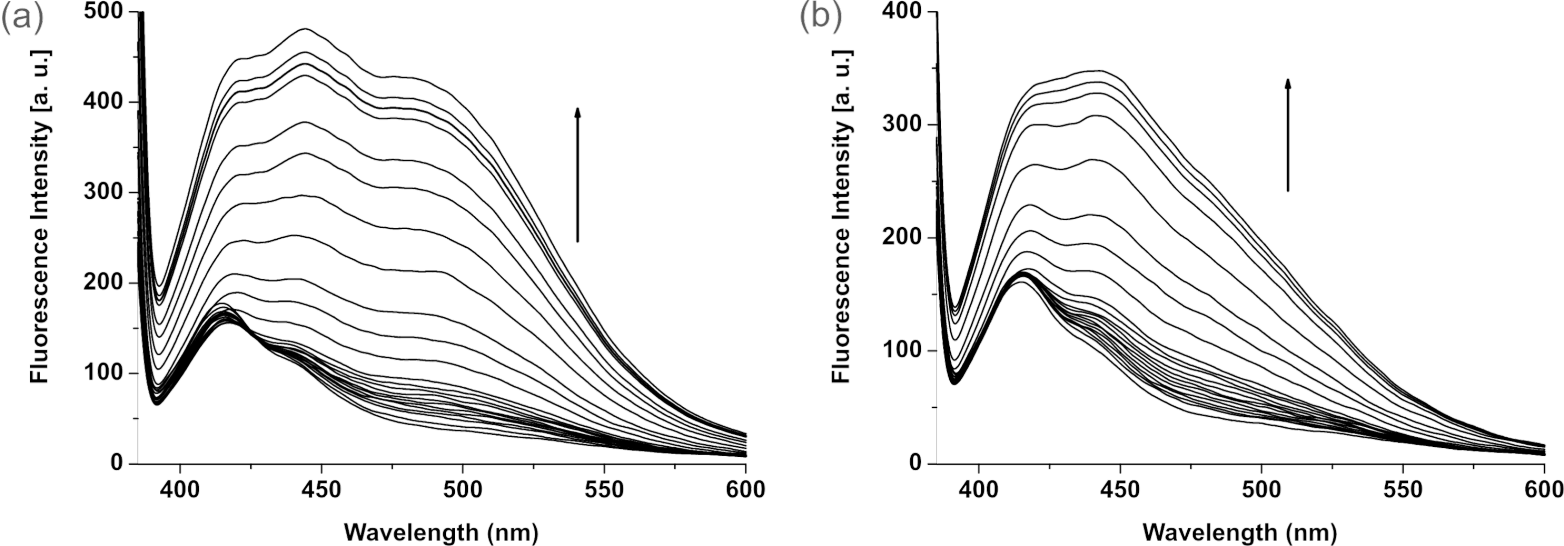
Change in emission of **1** (*c* = 4.31 × 10^−5^ M) upon gradual addition of tetrabutylammonium salts of (a) L-*N-*acetylvaline and (b) L-*N-*acetylalanine.

Figure 4a and Figure 4b show the change in emission of **1** (*c* = 4.31 × 10^−5^ M) upon increasing the quantity of tetrabutylammonium salts of L-*N*-acetylvaline and L-*N*-acetylalanine, respectively. The expected charge transfer in **1** upon complexation of valine salt was further established by performing similar fluorescence titration experiment with the receptor **2**, where the electron deficient urea motif is absent. In this case, no broad band at 492 nm was observed when the titration was carried out by gradual addition of the valine salt (Supporting Information File 1). This was also the case for the alanine salt. The other salts merely perturbed the emission of **2** and thus indicate the positive role of the urea motif in **1** for effective complexation of anionic substrates. This is in accordance with Hamilton’s observation [20].

**Figure 5:**
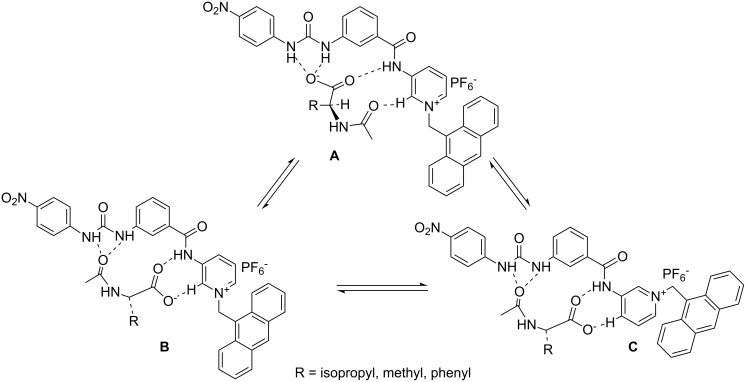
Suggested modes of binding of the amino acid salts into the open cleft of **1**.

**Figure 6:**
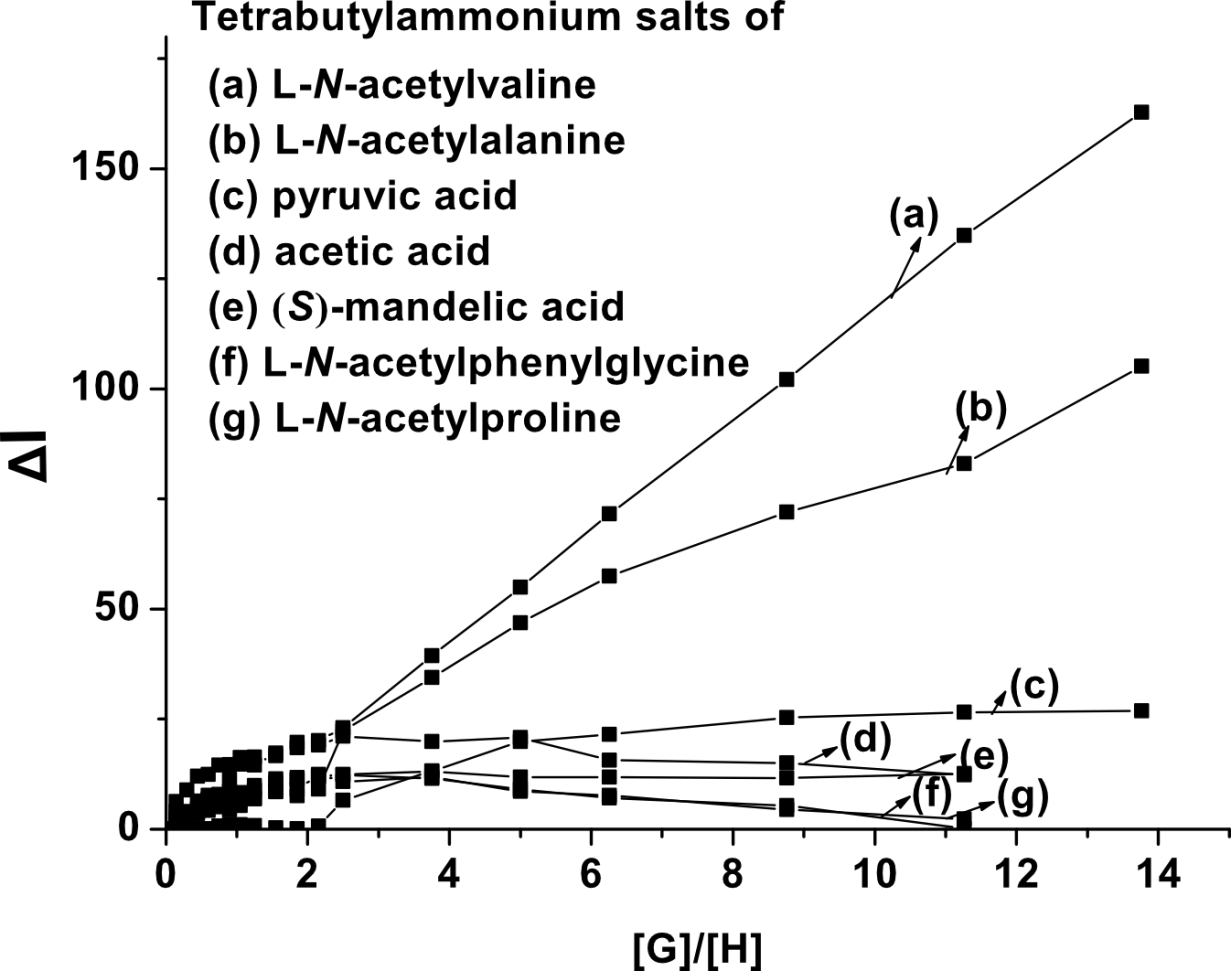
Fluorescence titration curves for **1** (*c* = 4.31 × 10^−5^ M) at 492 nm.

The different modes of emission (enhancing and quenching) of **1** in the presence of the different guests studied is believed to be due to the structural difference and hydrogen bonding abilities of the guests for which the PET process occurring between the amide-urea binding site and the excited state of anthracene is regulated in different ways. We presume that receptor **1** may follow any equilibrium-binding mode **A**, **B** or **C** with valine, alanine and phenylglycine salts in solution as shown in Figure 5. This is also true for the mandelate, pyruvate and proline salts. Relevance of the suggested modes in Figure 5 was followed from the change in chemical shift of the key protons of **1** in the ^1^H NMR upon complexation (see Figure 2) as well as from Hamilton’s observation on related systems [3]. In the interaction process, the stoichiometry of the complexes was 1:1 as confirmed by Job plots (Supporting Information File 1). In addition, the break in the titration curves at [G]/[H] = 1 in Figure 6 also supports this stoichiometry. A closer look at the curves for L-*N*-acetylvaline and L-*N-*acetylalanine salts in Figure 6 shows that there are breaks at [G]/[H] = 1 within the range of 3 equivalents of the added guest. Further addition causes a steady increase in emission and reaches saturation only when 20 to 30 equivalent amounts of the guest are added (Supporting Information File 1). This result suggests that in the presence of a large excess of the L-*N*-acetylvaline and L-*N-*acetylalanine salts that complexes with higher order stoichiometries are being formed in solution. The almost linear nature of the titration curves for all other guests except L-*N*-acetylvaline and L-*N-*acetylalanine salts at 492 nm (Figure 6) corroborated the weak interaction.

**Figure 7:**
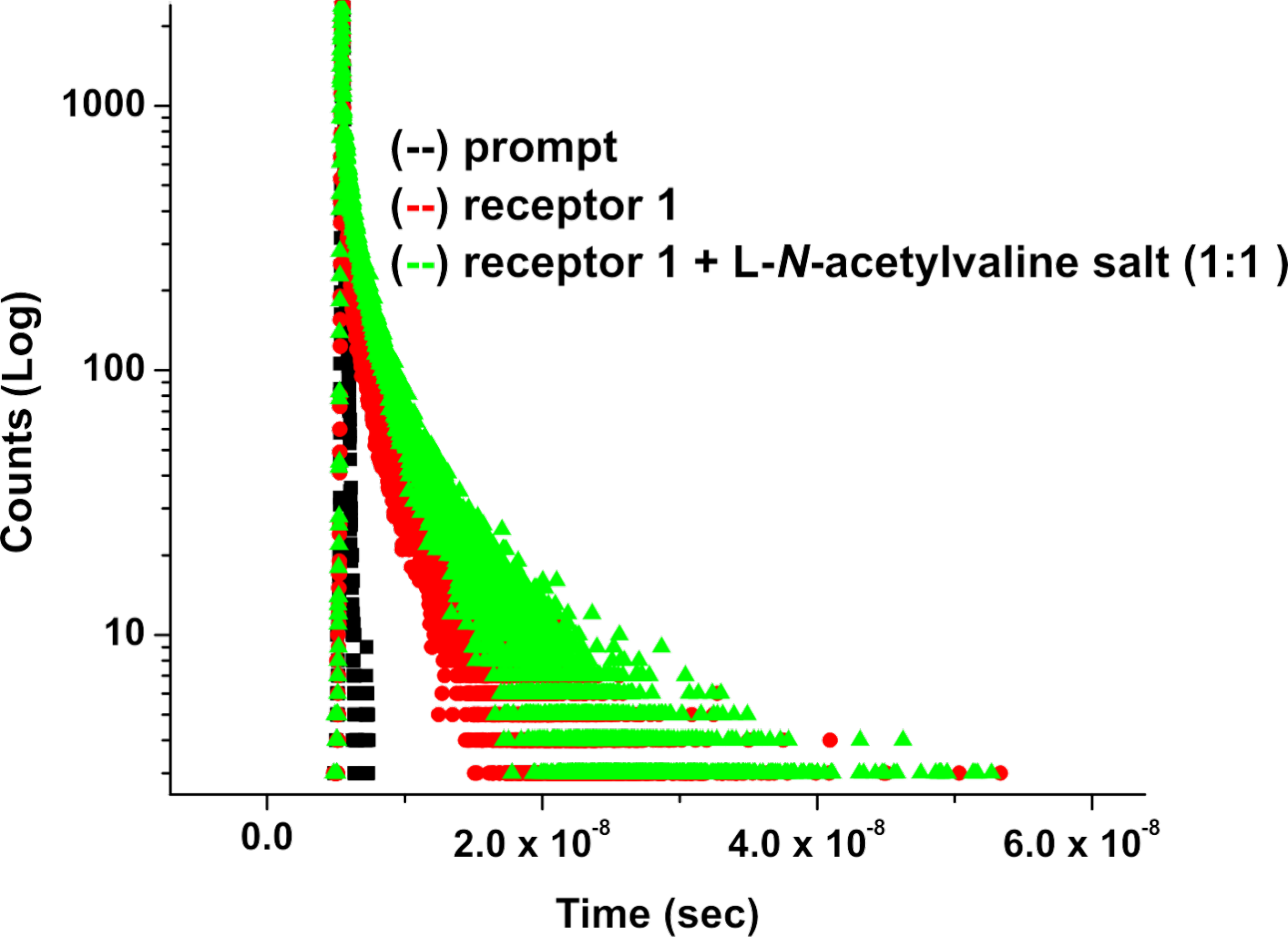
Fluorescence decays (at λ_max_ = 420 nm) of receptor **1** upon the addition of 1 equiv of L-*N*-acetylvaline salt ([H] = 4.71 × 10^−5^ M, [G] = 9.42 × 10^−4^ M) in CH_3_CN.

The time resolved emissions for **1** and **2** upon excitation at 370 nm were then studied to have an insight into the interaction process. The emission decay profile of **1** monitored at 420 nm could be fitted bi-exponential with two constants τ_1_ = 0.46 ps (100%), τ_2_ = 2.59 ns (0%). The faster decay component (0.46 ps) is either due to a very short-lived species or an artifact, or for tunneling of extra energy to the bulk by a non-radiative pathway [29–30]. The emitting species with a life time of 2.59 ns is real. However, in the presence of 1 equiv of L-*N-*acetylvaline salt, the decay profile followed a tri-exponential fitting that indicated three emitting species with life times τ_1_ = 1 ns (0.02%), τ_2_ = 4.23 ns (0.02%) and τ_3_ = 0.62 ps (99.96%) (Figure 7). Among these, the fast decay component 0.62 ps could be attributed to the formation of a hydrogen-bonded short-lived species where presumably intramolecular charge transfer between anthracene and nitrophenyl urea as represented in Figure 4a, takes place. This component coexists with the other components contributing large preexponential factor to the total fluorescence. This finding was not observed with L-*N-*acetylalanine or L-*N-*acetylproline salts (Table 1). Neither was this observed when the emission decay of **2** was monitored at 418 nm in the presence of L-*N-*acetylvaline salt upon excitation at 370 nm. This observation is thus noteworthy since it distinguishes the L-*N-*acetylvaline salt in the present study form the others with receptor **1**.

**Table 1:** Fluorescence decay times (τ), and preexponential factors for **1** and **2** in CH_3_CN.

Receptor in presence and absence of guest	Fluorescence decay time τ (preexponential factor)

**1**	τ_1_ = 0.46 ps ( 100%), τ_2_ = 2.59 ns ( 0%) ; (χ^2^ = 1.43)
**1** + 1 equiv L-*N*-acetylvaline salt	τ_1_ = 1 ns (0.02%), τ_2_ = 4.23 ns (0.02%), τ_3_ = 0.62 ps (99.96%); (χ^2^ = 1.08)
**1** + 1 equiv L-*N*-acetylalanine salt	τ_1_ = 5.0 ps (58.83%), τ_2_ = 2.75 ns (41.17%); (χ^2^ = 1.03)
**1** + 1 equiv L-*N*-acetylproline salt	τ_1_ = 2.5 ps (85.56%), τ_2_ = 2.76 ns (14.44%); (χ^2^ = 1.15)
**2**	τ_1_ = 0.58 ps (99.98%), τ_2_ = 3.92 ns (0.02%); (χ^2^ = 1.54)
**2** + 1 equiv L-*N*-acetylvaline salt	τ_1_ = 0.63 ps (99.95%), τ_2_ = 3.87 ns (0.05%); (χ^2^ = 1.72)
**2** + 1 equiv L-*N*-acetylalanine salt	τ_1_ = 2.52 ps (65.38%), τ_2_ = 3.84 ns (34.62%); (χ^2^ = 1.69)
**2** + 1 equiv L-*N*-acetylproline salt	τ_1_ = 0.49 ps (100%), τ_2_ = 4.18 ns (0%); (χ^2^ = 1.36)

**Figure 8:**
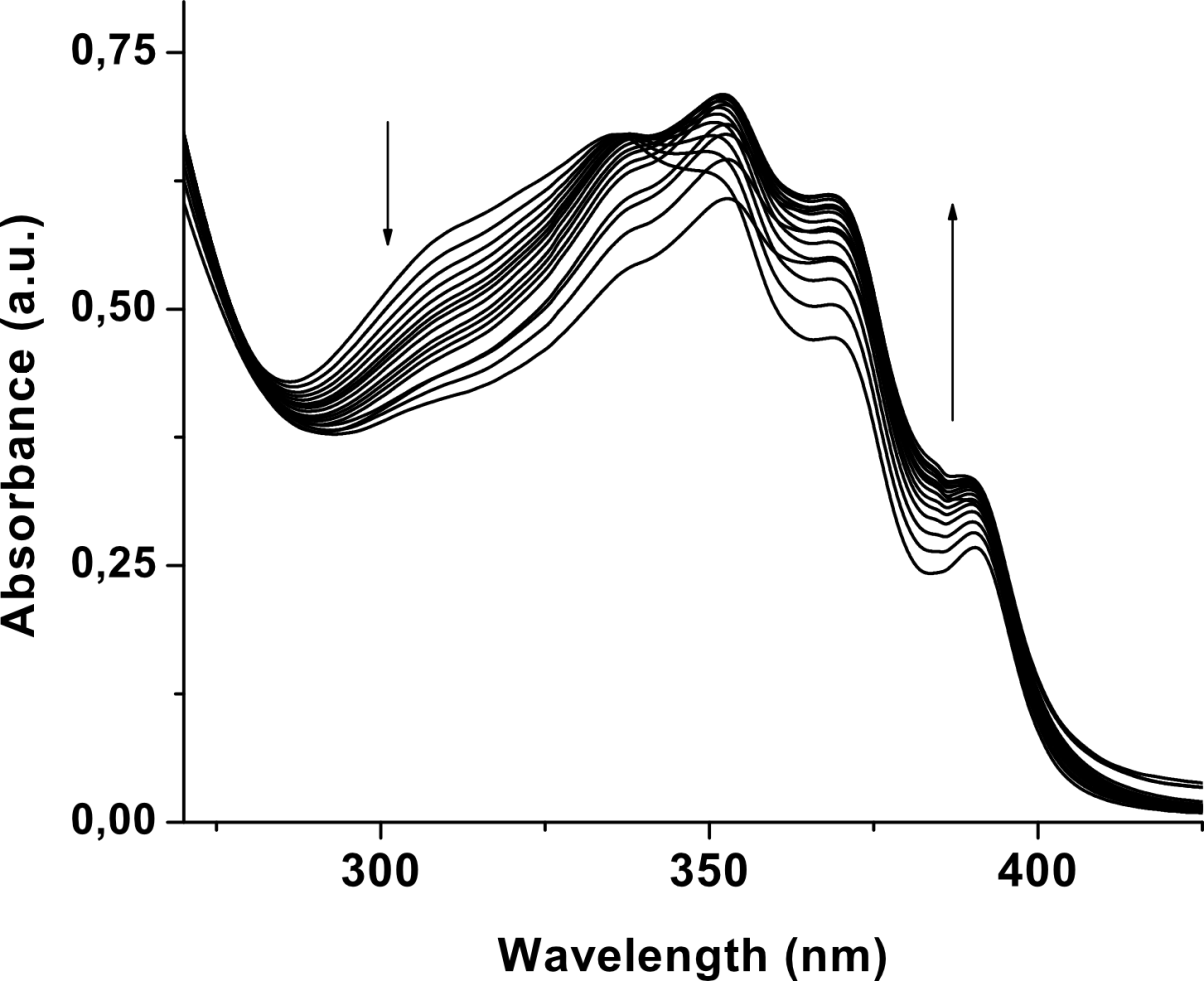
Change in absorbance of **1** (*c* = 4.31 × 10^−5^ M) upon gradual addition of tetrabutylammonium salts of L-*N-*acetylvaline.

Concurrently, the ground state binding properties of **1** in CH_3_CN were evaluated by UV–vis studies. The absorption spectrum of **1** (*c* = 4.31 × 10^−5^ M) in the absence of anions showed a structured band with maximum intensity at 341 nm. Upon titration with the salt of L-*N-*acetylvaline the ground state of **1** was affected significantly and the absorption was weakly red shifted because of recognition of the anion and a distinctive isosbestic point was observed at 340 nm (Figure 8). Similar observations were noted with the other guests (Supporting Information File 1). The stoichiometry of the interaction process in the ground state was also 1:1 as established from the break in the titration curves as well as Job plots [31] (Supporting Information File 1). In the titration curves, downward running of the titration curves in few cases indicates that as a greater excess of the guest anion is added, the 1:1 host–guest complex is assumed to be disrupted and anions begin to bind individually to the pyridinium amide and urea binding sites, rather than in a cooperative manner. Thus, in principle, equilibrium complexes of multiple stoichiometries can be generated in solution. This behavior depends on the anions [32] and also on the concentrations of the host and guest used at which the titration experiments are monitored.

Analysis of the emission data provided the association constants (*K*_a_), reported in Table 2 [33]. For determining binding constant values for L-*N-*acetylvaline and L-*N-*acetylalanine salts, we considered the emission data up to the addition of 13 equivalents of the guests added, since in the presence of large excess of such guests complex stoichiometries were noted. From Table 2 it can be seen that receptor **1** exhibits higher binding constant values for L-*N*-acetylvaline in the series. At this moment, we believe that it is due to structural aspects as well as to the hydrogen bonding capacity of the valine salt. Furthermore, to establish the hydrogen bonding influence of the acylamino and hydroxy groups of the amino and hydroxy acids respectively, we determined the binding constant for the acetate anion. This was found to be slightly less when compared to all the other guests. Pyruvate with carbonyl at the α-position did not produce any marked change in emission of **1**. We also determined the binding constants for **2** with the same anions in CH_3_CN by fluorescence (Table 2) and the values were found to be less compared to the values for **1**. This is in accordance with Hamilton’s observation [3] and indicates the role of the urea motif in the binding event.

**Table 2:** Association constants (*K*_a_) in CH_3_CN from fluorescence measurements.

Guest^a^	Receptor **1** *K*_a_ [M^−1^]^e^	Receptor **2** *K*_a_ [M^−1^]^e^

L-*N*-acetylvaline	1.87 x 10^4^; R = 0.998^c^	1.30 x 10^3^; R = 0.988^c^
L-*N*-acetylalanine	2.60 x 10^3^; R = 0.995^d^	6.50 x 10^2^; R = 0.993^c^
L-*N*-acetylproline	1.38 x 10^3^; R = 0.993^c^	—^b^
L-*N*-acetylphenylglycine	1.31 x 10^3^; R = 0.989^c^	—^b^
acetate	2.20 x 10^3^; R = 0.997^c^	1.70 x 10^3^; R = 0.998^c^
(*S*)-mandelate	1.60 x 10^3^; R = 0.995^c^	—^b^
pyruvate	—^b^	—^b^

^a^tetrabutylammonium salts were used; ^b^not determined due to irregular change; ^c^determined at 414 nm; ^d^determined at 492 nm; ^e^error ≤ 10%.

**Figure 9:**
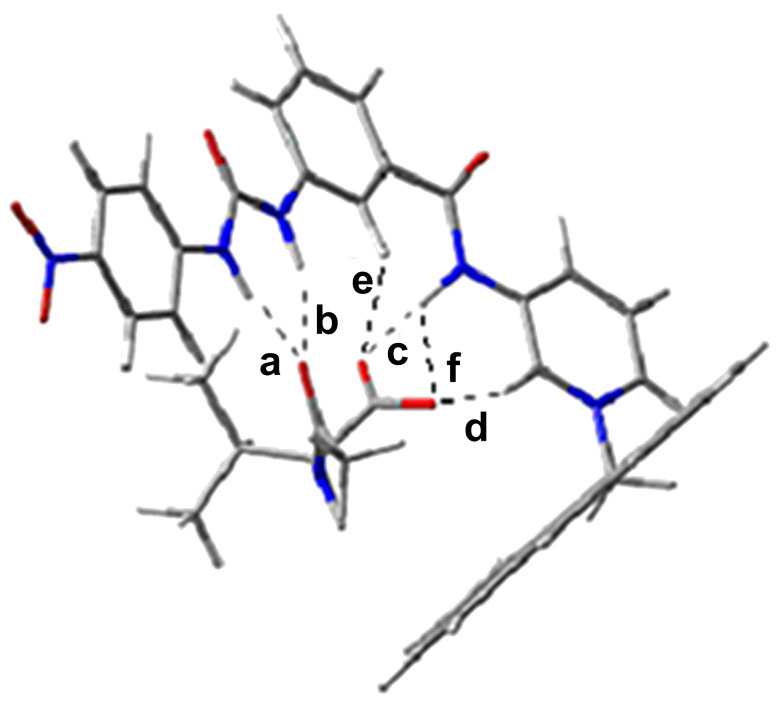
DFT optimized geometry of the complex of **1** with L-*N-*acetylvaline carboxylate salt [a = 1.93 Å, b = 1.99 Å, c = 1.64 Å, d = 1.97 Å, e = 2.60 Å and f = 2.69 Å].

In order to realize the conformational behavior and the reactivity of **1** towards hydrogen bonding with the anionic guests as noted in Table 2, we carried out detailed DFT calculations. The Gaussian-03 package [34] and GAMESS-US suite [35] (version April 11, 2008) was used for the calculations and the MO figures were obtained using the MaSK software [36]. DFT calculations were performed in the gas phase on the two different conformations of **1** using the 6-311G^**^ basis set [37] and the popular b3LYP functional [38–39]. Similar calculations were also carried out on all the guests given in Table 2. For all the compounds, parameters such as global electrophilicity (ω), global electronegativity (χ), global hardness (η), dipole moment (µ) and the energies of HOMO and LUMO are also reported (Table S1; Supporting Information File 1) from their DFT optimized structures [34–36]. The orientation of the binding sites in both **1A** and **1B** are displayed in Figure S15 (Supporting Information File 1). Of the two possible conformations of receptor **1** (Figure S15), **1B** is found to be more stable than **1A** by 2.82 kcal/mol. Therefore, we may reasonably expect that in solution both conformers may remain in equilibrium. However, conformation **1A** may form a more stable hydrogen-bonded complex with guests, owing to formation of a greater number of hydrogen bonds with the guests. For example, Figure 9 demonstrates the hydrogen-bonded complex of **1A** with *N*-acetyl-L-valine carboxylate, where both the urea and pyridinium motifs are cooperatively involved in bonding.

However, interaction between the electrophile and the nucleophile can be quantified in terms of the electrophilicity index (ω) of individual species involved in the process. Although ω of structure **1A** is found to be only slightly different from that of **1B** (Supporting Information File 1), it is obvious that structure **1** has an affinity for anionic guests. As can be seen from Table S1 (Supporting Information File 1), guests such as the salts of valine, phenylglycine and mandelic acid exhibit the lowest values of ω among all anionic guests considered. This indicates that these guests have higher nucleophilic character compared to the other guests studied. Therefore, receptor **1**, in principle, will show strong binding for these salts. Experimental results support this observation (see Table 2). Furthermore, to rationalize the preferential mode of interaction of the carboxylate guests with the two different binding domains (urea and pyridinium sites) of receptor **1**, we further calculated Fukui functions [40] for nucleophilic attack (*f*_k_^+^) at these two sites. The larger the value, the greater is the reactivity of the site towards a nucleophile. Between the two sites of **1**, pyridinium amide exhibits a value of 0.5384, which is significantly greater than the value obtained for urea site (0.0021) and thus binding of the carboxylate part of all the guests will take place preferentially at the pyridinium site instead of urea (see structures **B**/**C** in Figure 5).

In conclusion, we have designed and synthesized a new fluororeceptor **1**, which is able to bind α-acylamino as well as hydroxy acids with moderate binding constant values. The receptor clearly distinguishes L-*N-*acetylvaline and L-*N-*acetylalanine salts from the other anionic substrates studied by exhibiting different modes of emission. The appearance of charge transfer band and three decay components in time resolved spectroscopy upon complexation of L-*N-*acetylvaline salt are the distinct features in the present study to distinguish it from the other anions examined. Further progress in this direction is underway in our laboratory.

## Supporting Information

Synthesis and characterization data for **1** and **2**, general procedure for fluorescence and UV titrations, change in absorption and fluorescence spectra, Job plots of receptor **1** with few selected guests, change in emission of **2** in the presence of few selected anions, UV titration curves for **1**, selected binding constant curve for **1**, change in emission of **1** upon dilution with solvent, titration curves for **1** with valine and alanine salts, DFT optimized geometry of **1** and MO energies, global hardness, global electronegativity, global electropositivity, dipole moment etc. are available.

File 1 Detailed experimental data for **1** and **2**.
