# Supplementary material for: Anthracene appended pyridinium amide–urea conjugate in selective fluorometric sensing of L-N-acetylvaline salt
Source: Beilstein J Org Chem. 2010 Dec 21;6:1211–8. doi: 10.3762/bjoc.6.139 (PMC3028924; doi:10.3762/bjoc.6.139)

Supporting Information
for

Anthracene appended pyridinium amide–urea conjugate in selective fluorometric sensing of *N*-acetyl-L-valine salt

Kumaresh Ghosh*, Tanmay Sarkar and Asoke P. Chattopadhyay

Address: Department of Chemistry, University of Kalyani, Kalyani-741235, India, Fax +91-33-25828282

Email: Kumaresh Ghosh - ghosh_k2003@yahoo.co.in

*Corresponding author

Detailed experimental data for **1** and **2**

Synthesis

3-(3-(4-nitrophenyl)ureido)-*N*-(pyridin-3-yl)benzamide 5

To the solution of 3-aminopyridine (0.5 g, 5.3 mmol) in dry CH_2Cl_2 (25 mL), Et_3N (0.767 ml, 5.3 mmol) was added followed by the addition of 3-nitrobenzoyl chloride (1.48 g, 7.9 mmol) under a nitrogen atmosphere. The reaction mixture was stirred at room temperature for 3 h. After completion of the reaction, solvent was evaporated and the residue extracted with a $CHCl_3$-MeOH mixture (3 x 30 mL). The combined extracts were washed with water, dried over anhydrous Na_2SO_4 and concentrated in vacuo. The crude product was purified by silica gel column chromatography with 50% petroleum ether in ethyl acetate as eluent to afford the nitro compound **3** (237 mg, yield 52%).

Compound **3** (0.3 g, 1.2 mmol) was dissolved in ethyl acetate (30 mL) and $SnCl_2$ (1.3 g, 6.17 mmol) was added. The resulting solution was stirred for 4 h at room temperature. The reaction mixture was basified with aq. $NaHCO_3$ and extracted with ethyl acetate (3 x 30 mL). The combined extracts were dried over Na_2SO_4 and concentrated under reduced pressure to afford compound **4** (236 mg, yield 90%, mp 150-152 °C). Compound **4** was sufficiently pure enough to use in the next step without further purification.

Reaction of **4** (0.210 g, 0.985 mmol) with 4-nitrophenyl isocyanate (obtained by the reaction of 4-nitroaniline (0.136 g, 0.985 mmol) with triphosgene (0.292 g, 0.985 mg) in dry THF (20 mL) containing Et_3N (0.312 mL, 2.16 mmol)) gave compound **5** as a yellowish white solid (0.22 g, yield 60%); mp 224-226 °C; ^{1}H NMR (400 MHz, $CDCl_3$ containing one drop of d_6- DMSO) δ 9.74 (s, 1H, NH), 8.93 (s, 1H), 8.92 (s, 1NH), 8.64 (s, 1H), 8.36 (s, 1H, NH), 8.31 (d, 1H, J = 8 Hz) , 8.17 (d, 2H, J = 8 Hz), 7.90-7.86 (m, 2H), 7.64 (d, 2H J = 8 Hz), 7.63 (s, 1H), 7.43 (t, 1H, J = 8 Hz), 7.31 (t, 1H, J = 8 Hz); FTIR (υ in cm^{-1}, KBr): 3257, 3080, 2699, 1707, 1679, 1594, 1538, 1491 cm^{-1}; Anal. Calcd for $C_{19}H_{15}N_5O_4$: C, 60.47; H, 4.01; N, 18.56. Found: C, 60.58; H, 4.15; N, 18.61.

1-(Anthracen-9-ylmethyl)-3-(3-(4-nitrophenyl)urido)benzamido)pyridinium hexafluro phosphate(V) 1

Compound **5** (0.1g, 0.2.75 mmol) was dissolved in dry CH_3CN (20 mL) with warming. n 9-Chloromethylanthracene (0.066 g, 0.291 mmol.) was added and the reaction mixture heated under reflux for 3 days under a nitrogen atmosphere. During the reaction, chloride

salt **6** was precipitated. The salt was filtered and washed several times with CH_3CN. The chloride salt **6** was dissolved in aq. MeOH with warming and chloride ion was exchanged by the addition of NH_4PF_6. The yellow precipitate of **1** was collected by filtration. Repeated recrystalization of the salt **1** from a $CHCl_3/CH_3OH$ mixture solvent gave the pure product (0.1 g, yield 85%); mp 188 - 190 °C; ^{1}H NMR (400 MHz, d_6-DMSO) δ 11.10 (s, NH, 1H), 9.52 (s, NH, 1H), 9.30 (s, NH, 1H), 9.20 (s, 1H), 8.96 (s, 1H), 8.80 (d, 1H, *J* = 8 Hz), 8.74 (d, 1H, *J* = 8 Hz), 8.43 (d, 2H, *J* = 8 Hz), 8.27 (d, 2H, *J* = 8 Hz), 8.20 (d, 2H, *J* = 8 Hz), 8.12 (t, 1H, *J* = 8 Hz), 8.01 (br s, 1H), 7.95 (br s, 1H), 7.72 – 7.62 (m, 6H), 7.51 – 7.43 (m, 2H), 7.00 (s, 2H); ^{13}C NMR (d_6-DMSO, 100 MHz): 166.3, 162.1, 151.8, 146.0, 141.0, 139.5, 138.7, 135.0, 134.1, 133.7, 131.3, 131.1, 130.9, 129.4, 129.0, 128.2, 128.1, 125.6, 125.0, 123.1, 122.5, 121.6, 121.5, 117.8, 117.5, 56.3; FTIR (υ in cm^{-1}, KBr):3138, 2918, 2849, 1652, 1606, 1593, 1558, 1402; *m/z* (ES^+): 568.2 $[(M- PF_6^-)]^+$.

1-(Anthracen-9-ylmethyl)-3-(-3-butyramidobenzamido)pyridium hexafluoro phosphate (V) 2

To the solution of **4** (0.3 g, 1.4 mmol) in dry CH_2Cl_2 (25 mL), Et_3N (0.20 mL, 1.4 mmol) was added followed by *n*-butyryl chloride (0.18 mL, 1.7 mmol) under a nitrogen atmosphere. The reaction mixture was stirred at room temperature for 4 h. After completion of the reaction, solvent was evaporated and the residue extracted with a $CHCl_3$-MeOH mixture (3 x 30 mL). The combined extracts were washed with water, dried over anhydrous Na_2SO_4 and concentrated in vacuo. The crude product was purified by silica gel column chromatography with 30% petroleum ether in ethyl acetate as eluent to afford the compound **7** (0.290 g, yield 75%); mp 98 °C; ^{1}H NMR (400 MHz, d_6-DMSO) δ 9.85 (s, 1H), 9.28 (s, 1H), 8.90 (s, 1H), 8.33 – 8.29 (m, 2H), 8.06 (s, 1H), 7.88 (d , 1H, *J* = 8 Hz), 7.67 (d, 1H, *J* = 8 Hz), 7.41 - 7.37 (m, 1H), 7.30 (t, 1H, *J* = 8 Hz), 2.37 (t, 2H, *J* = 8 Hz), 1.78 – 1.72 (m, 2H), 1.00 (t, 3H, *J* = 8 Hz); FT-IR (υ in cm^{-1}, KBr): 3470, 3327, 1658, 1629, 1582, 1525, 1421.

Compound **7** (0.1 g, 0.353 mmol) was dissolved in dry CH_3CN (20 mL) with warming. 9-Chloromethylanthracene (0.087 g, 0.388 mmol) was added and the reaction mixture was heated under reflux for 2 days under a nitrogen atmosphere. During the reaction chloride salt precipitated and was collected by filtration. The salt was washed several times with

CH_3CN. Anion exchange of chloride salt was performed in aq. MeOH with warming by the addition of NH_4PF_6. The yellow precipitate of **2** was collected by filtration. Repeated recrystallization of **2** from a $CHCl_3/CH_3OH$ mixture solvent gave the pure product (0.1 g, yield 90%), mp 222-224 °C; ^{1}H NMR (400 MHz, d_6-DMSO) δ 11.09 (s, 1H), 10.09 (s, 1H), 9.28 (s, 1H), 8.96 (s, 1H), 8.82 (d, 1H, *J* = 6 Hz), 8.73 (d , 1H, *J* = 8 Hz), 8.43 (d, 2H, *J* = 8 Hz), 8.28 (d, 2H, *J* = 8 Hz) , 8.14-8.09 (m, 2H), 7.77 (d, 1H, *J* = 8 Hz), 7.71-7.63 (m, 4H) , 7.51 (d, 1H, *J* = 8 Hz) , 7.43 (t, 1H, *J* = 8 Hz), 6.99 (s, 2H), 2.28 (t, 2H, *J* = 7.6 Hz), 1.62 – 1.51 (m, 2H), 0.92 (t, 3H, *J* = 7.6 Hz); ^{13}C NMR (100 MHz , d_6-DMSO) δ 171.5, 166.4, 139.7, 139.6, 138.9, 135.1, 134.1, 133.6, 131.4, 131.2, 131.0, 129.5, 128.9, 128.3, 125.8, 123.2, 122.9, 122.1, 121.6, 118.4, 56.4, 38.2, 18.4, 13.6; FT-IR (υ in cm^{-1}, KBr) 3568, 3402, 3099, 2930, 1682, 1626, 1592, 1550, 1500, 1450.

2. General method for UV and fluorescence titrations:

Stock solutions of the hosts were prepared in CH_3CN containing 0.01% DMSO and 2 ml of the individual host solution was placed in a cuvette. The solution was irradiated at the excitation wavelength 370 nm maintaining the excitation and emission slits 12 and 10, respectively. Upon addition of the guest, dissolved in CH_3CN, the change in fluorescence emission of the host was observed. The corresponding emission values during titration were noted and used for the determination of binding constant values.

Similarly, in the UV titration, 2 ml of the individual host was placed in a cuvette and guest was gradually added to the host solution.

3. Fluorescence spectra

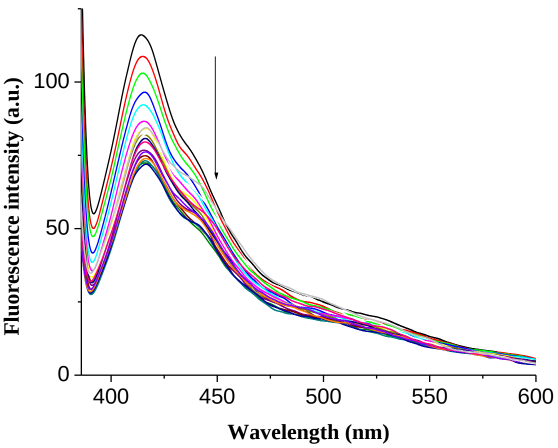

Figure S1: Change in emission of **1** (c = 4.31 × 10^{-5} M) upon gradual addition of tetrabutylammonium salt of L-*N*-acetylproline.

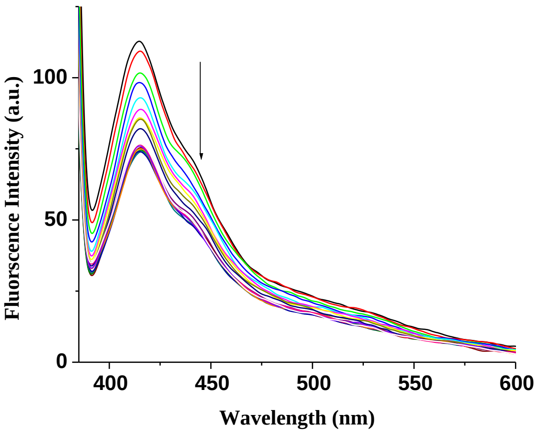

Figure S2: Change in emission of **1** (c = 4.31 × 10^{-5} M) upon gradual addition of tetrabutylammonium salt of (S)-mandelate.

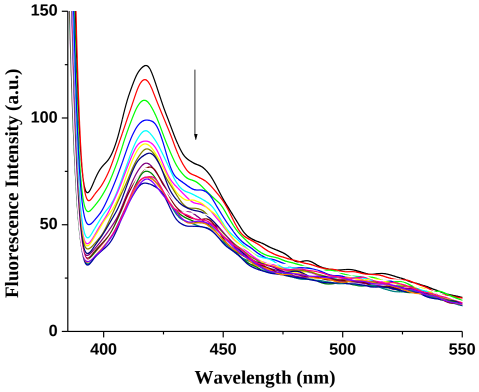

Figure S3: Change in emission of **1** (c = 4.31 × 10^{-5} M) upon gradual addition of tetrabutylammonium salt of (S)-acylamino phenyl glycine.

4. Absorption spectra

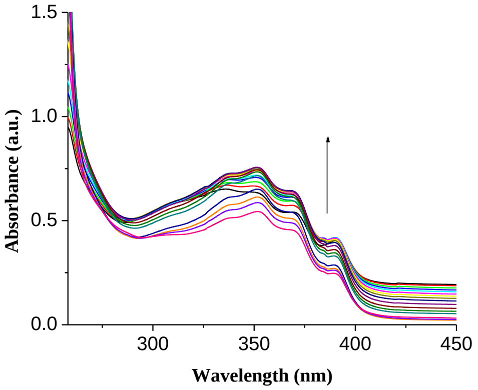

Figure S4: Change in absorbance of **1** (c = 4.31 × 10^{-5} M) upon gradual addition of tetrabutylammonium salt of L-*N*-acetylalanine.

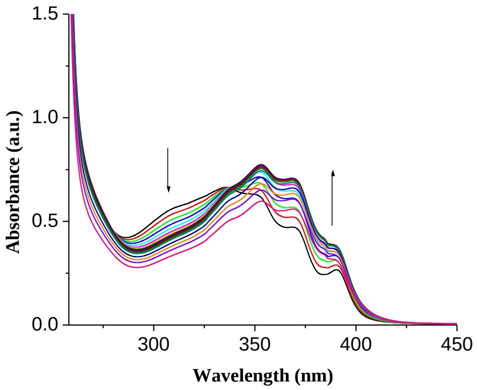

Figure S5: Change in absorbance of **1** (c = 4.31 × 10^{-5} M) upon gradual addition of tetrabutylammonium salt of L-*N*-acetylproline.

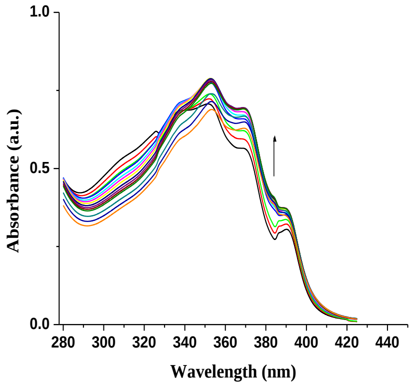

Figure S6: Change in absorbance of **1** (c = 4.31 × 10^{-5} M) upon gradual addition of tetrabutylammonium salt of (S)-*N*-acetylphenylglycine.

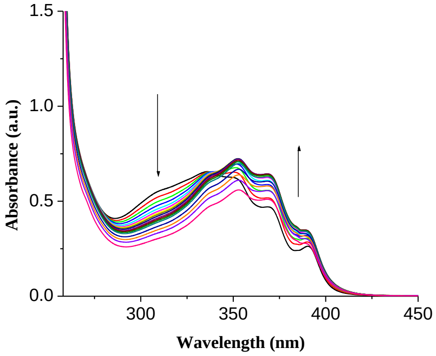

Figure S7: Change in absorbance of **1** (c = 4.31 × 10^{-5} M) upon gradual addition of tetrabutylammonium salt of (S)-mandelate.

5. UV Job plots

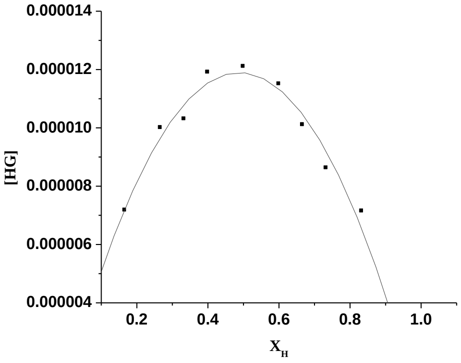

Figure S8: UV–vis Job plot of **1** with tetrabutylammonium salt of L-*N*-acetylalanine.

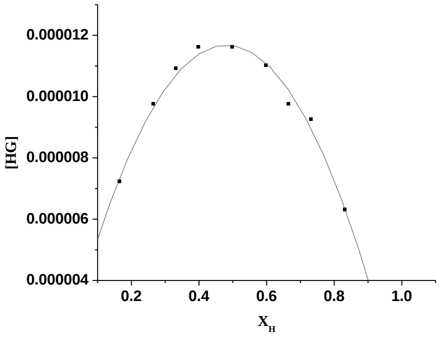

Figure S9: UV–vis Job plot of **1** with tetrabutylammonium salt of L-*N*-acetylvaline.

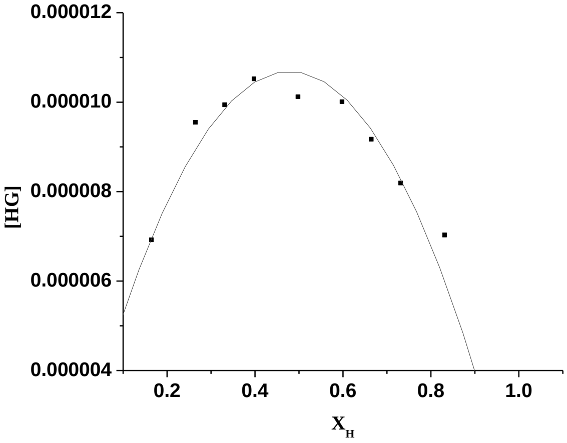

Figure S10: UV–vis Job plot of **1** with tetrabutylammonium salt of L-*N*-acetylproline.

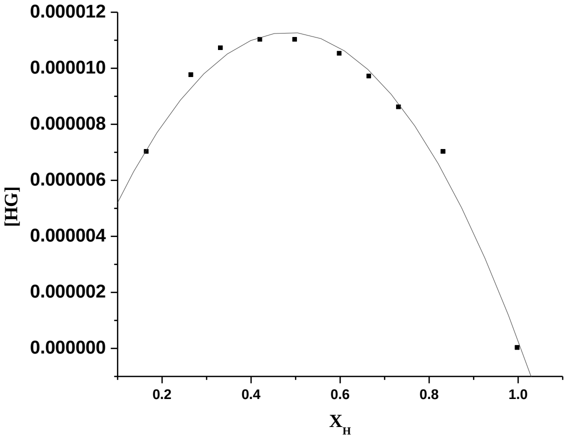

Figure S11: UV–vis Job plot of **1** with tetrabutylammonium salt of (*S*)-mandelic acid.

6. Fluorescence titration spectra for 2 with selected anionic guests

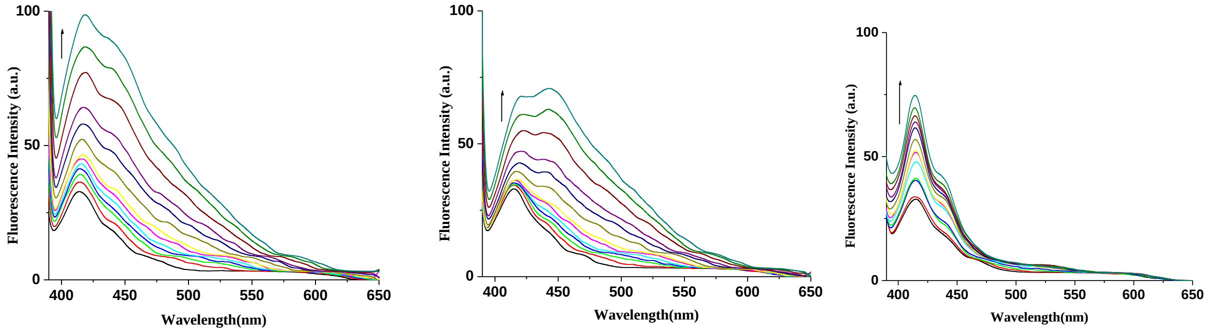

(a) (b) (c)

Figure S12: Change in emission of **2** ($c = 4.31 \times 10^{-5}$ M) upon gradual addition of tetrabutylammonium salts of (a) L-*N*-acetylvaline, (b) L-*N*-acetylalanine and (c) acetic acid.

7. UV titration curves for 1.

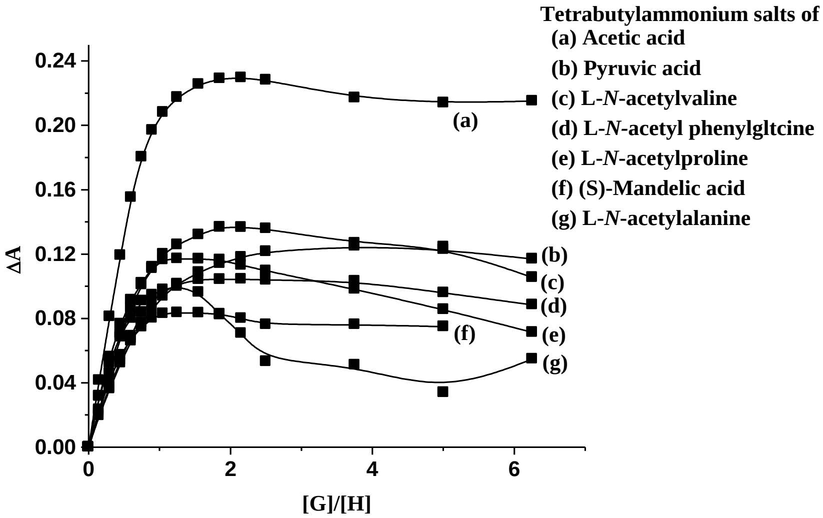

Figure S13: UV titration curves for **1** ($c = 4.31 \times 10^{-5}$ M) at 390 nm.

8. Binding constant curve for 1 with L-*N*-acetylvaline

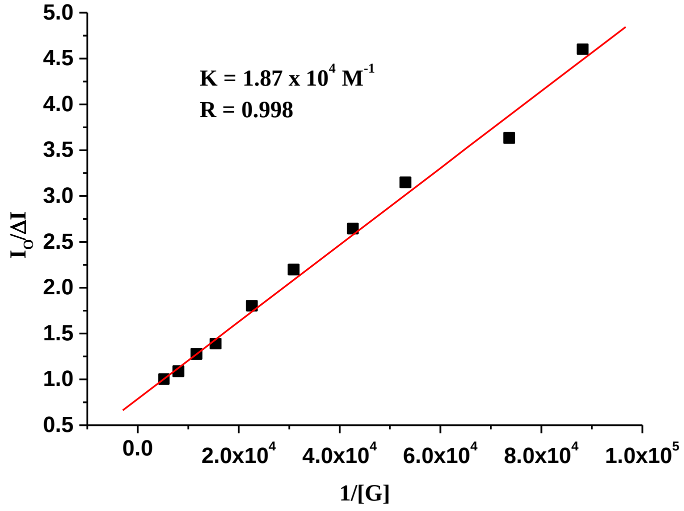

Figure S14: Binding constant curve for **1** with tetrabutylammonium salt of L-*N*-acetylvaline at 414 nm.

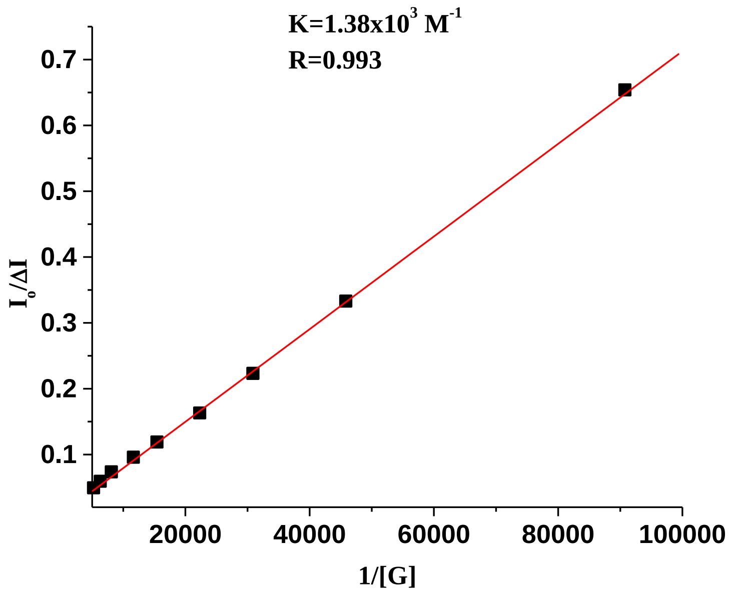

Figure S15: Binding constant curve for **1** with tetrabutylammonium salt of L-*N*-acetylproline at 414 nm.

Method for the determination of binding constant values:

The emission data were used for the determination of binding constant values. Binding constants were determined by using the expression $I_0/I_0\text{-}I = [\Phi_H/(\Phi_H - \Phi_C)](K_a^{-1}.Cg^{-1} + 1)$, where Φ_H and Φ_C are quantum yields for receptor and the hydrogen-bonding complex, respectively, at the selected wavelength, I_0 denotes the emission intensity of the free receptor at the specific wavelength and Cg is the concentration of the anionic guest. The measured emission I_0 /I_0-I as a function of the inverse of the anion concentration fits a linear relationship, indicating 1:1 stoichiometry of the receptor-anion complex. The ratio of the intercept to the slope was used to determine the binding constant K_a.

9. Change in emission of 1 upon dilution with the solvent.

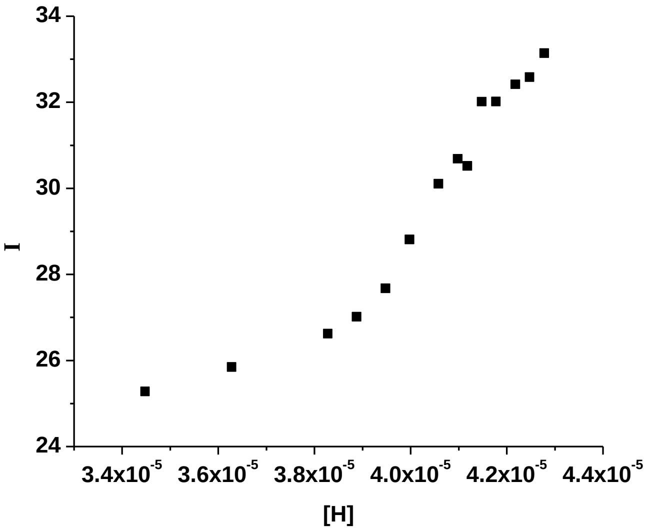

Figure S16: Change in emission of **1** ($c = 4.40 \times 10^{-5}$ M) upon gradual addition of CH_3CN.

10. Fluorescence titration curves for 1 with L-*N*-acetylvaline and L-*N*-acetylalanine carboxylates.

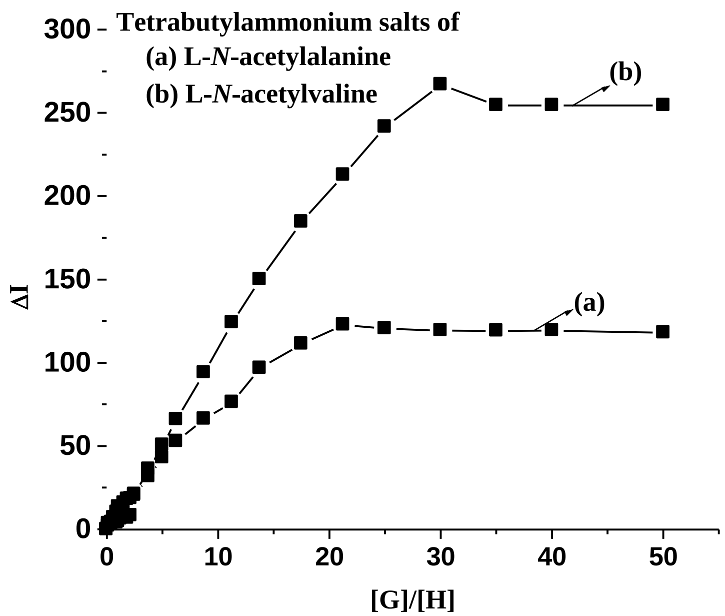

Figure S17: Fluorescence titration curves for **1** ($c = 4.31 \times 10^{-5}$ M) at 492 nm.

11. DFT optimized two different conformations of 1.

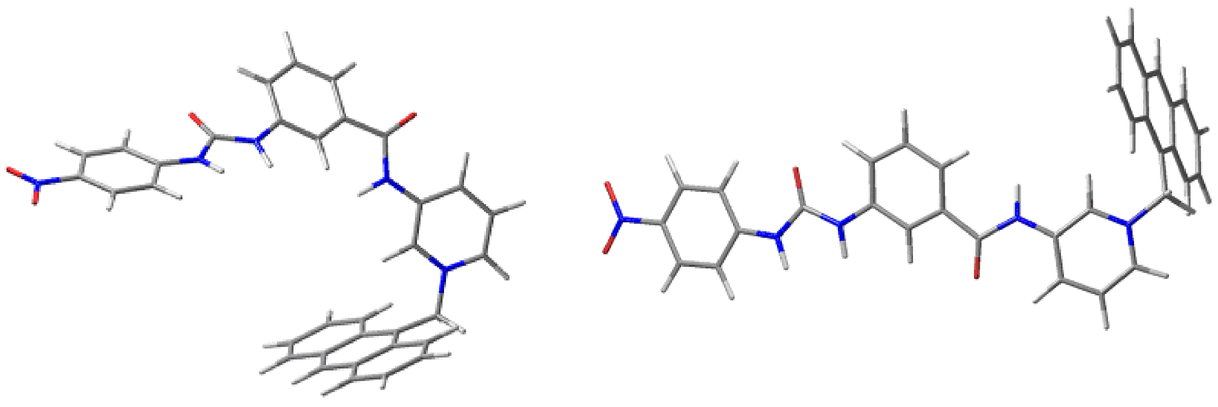

1A

1B

Figure S18: DFT optimized geometries of two conformations of **1**.

12. Energies of HOMO and LUMO, global electrophilicity (ω), global electronegativity (χ), global hardness (η), dipole moment values (μ) of receptor 1 and guests.

Table S1: Energies of HOMO and LUMO, global electrophilicity (ω), global electronegativity (χ), global hardness (η), dipole moment values (μ) of receptor 1

**Structures**	**E_{DFT} (au)**	**E_{HOMO} (au)**	**E_{LUMO} (au)**	**μ (D)**	**η**	**χ**	**ω**
**1A**	-1886.144	-0.3095	-0.2144	22.64	0.0951	0.2619	0.3608
**1B**	-1886.148	-0.3033	-0.2129	25.58	0.0904	0.2581	0.3683
L-*N*-acetylalanine[a]	- 476.003	-0.0529	0.1563	6.10	0.2092	-0.0517	0.0064
L-*N*-acetylvaline[a]	- 554.652	-0.0563	0.1505	6.09	0.2068	-0.0471	0.0054
L-*N*-acetylproline[a]	- 553.423	-0.0480	0.1528	6.59	0.2008	-0.0524	0.0068
L-*N*-acetylphenyl Glycine[a]	- 667.766	-0.0567	0.1188	6.92	0.1754	-0.0310	0.0027
Acetate[a]	- 228.576	-0.0071	0.2205	3.04	0.2276	-0.1067	0.0250
Pyruvate[a]	-341.944	-0.0286	0.1360	4.54	0.1646	-0.0537	0.0088
(S)-Mandelate[a]	- 534.952	-0.0577	0.1185	7.26	0.1762	-0.0304	0.0026

[a]Tetrabutylammonium salts were used.

^{1}H NMR (400 MHz, d_6-DMSO)

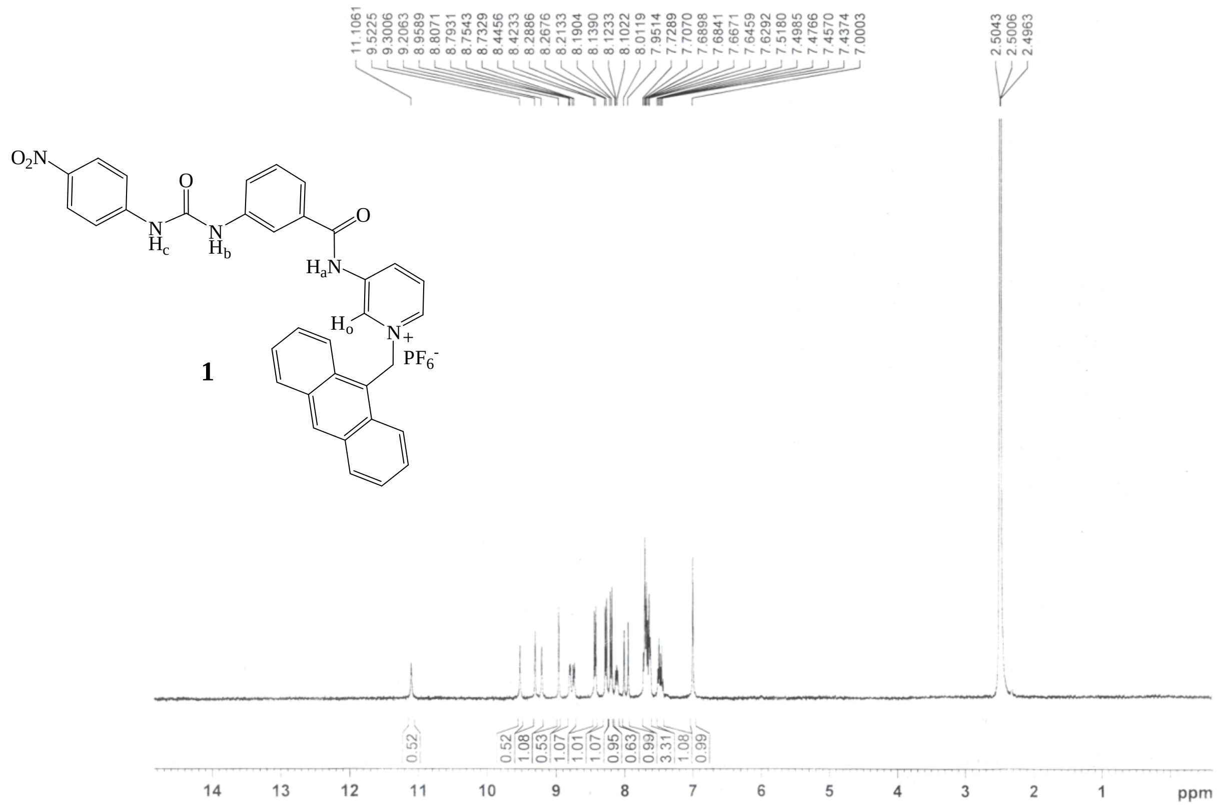

^{13}C NMR (100 MHz, d_6-DMSO)

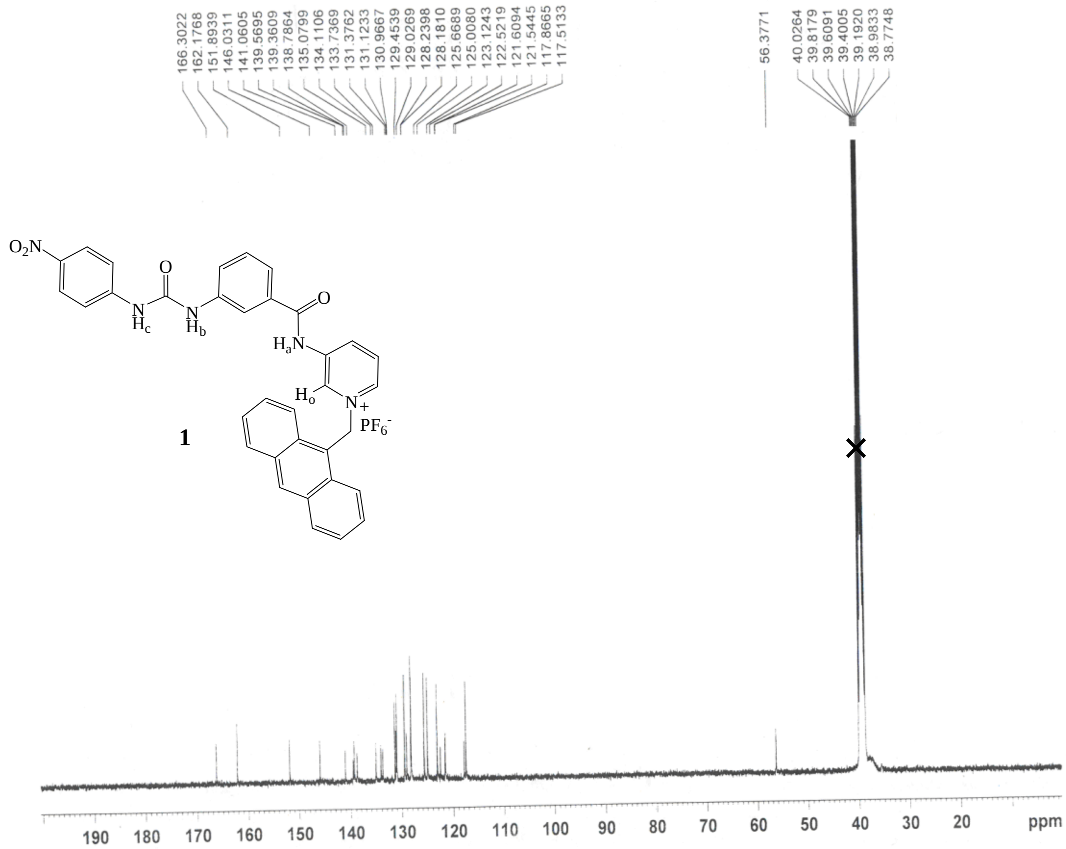

Mass spectra of compound 1:

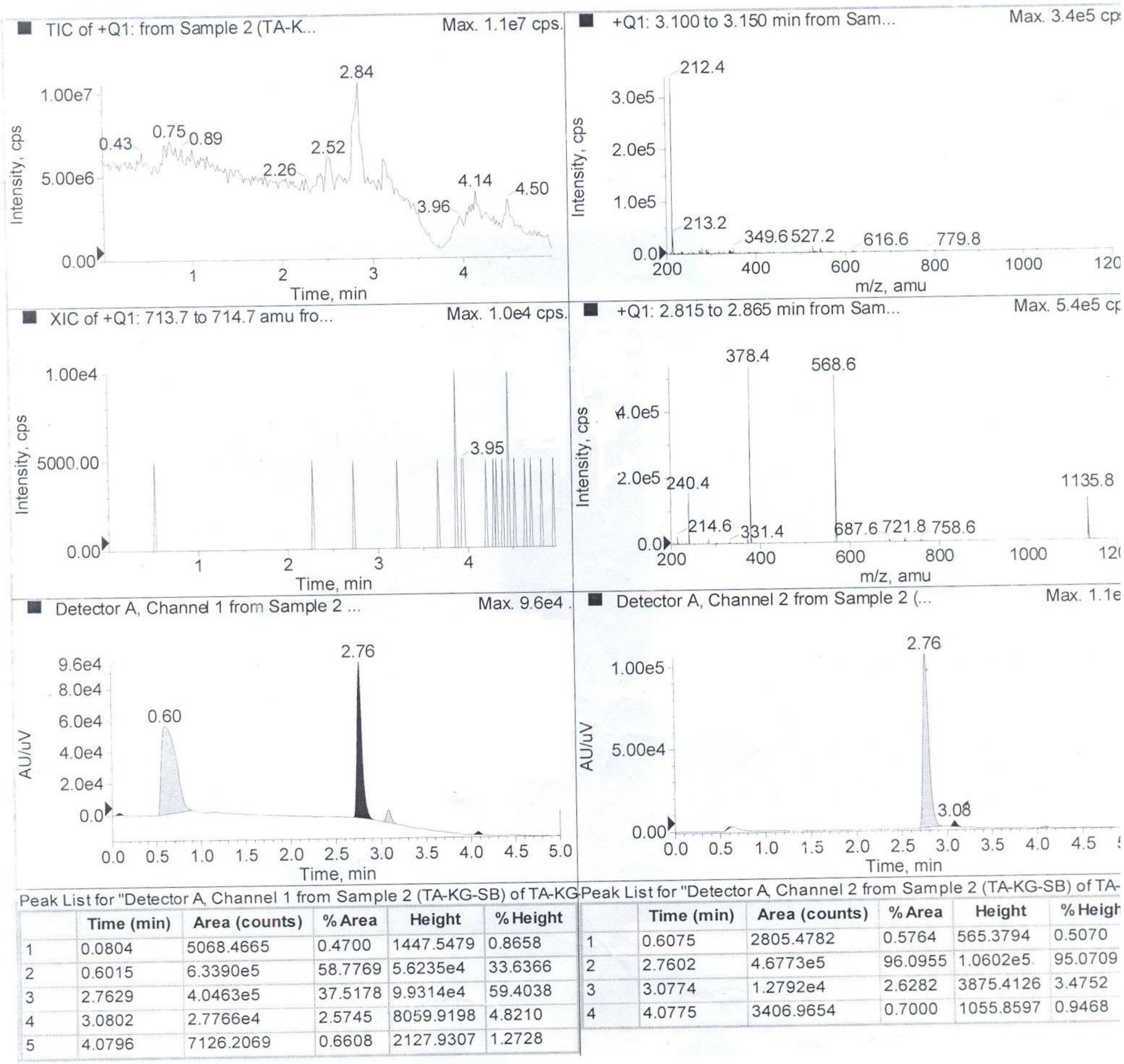

Peak List for "Detector A, Channel 1 from Sample 2 (TA-KG-SB) of TA-KG

	Time (min)	Area (counts)	% Area	Height	% Height
1	0.0804	5068.4665	0.4700	1447.5479	0.8658
2	0.6015	6.3390e5	58.7769	5.6235e4	33.6366
3	2.7629	4.0463e5	37.5178	9.9314e4	59.4038
4	3.0802	2.7766e4	2.5745	8059.9198	4.8210
5	4.0796	7126.2069	0.6608	2127.9307	1.2728

Peak List for "Detector A, Channel 2 from Sample 2 (TA-KG-SB) of TA-

	Time (min)	Area (counts)	% Area	Height	% Heigh
1	0.6075	2805.4782	0.5764	565.3794	0.5070
2	2.7602	4.6773e5	96.0955	1.0602e5	95.0709
3	3.0774	1.2792e4	2.6282	3875.4126	3.4752
4	4.0775	3406.9654	0.7000	1055.8597	0.9468

```
LCMS Reaction Monitori

Channel 1 at wavelengt
Channel 2 at wavelengt
```

^{1}H NMR (400 MHz, d_6-DMSO)

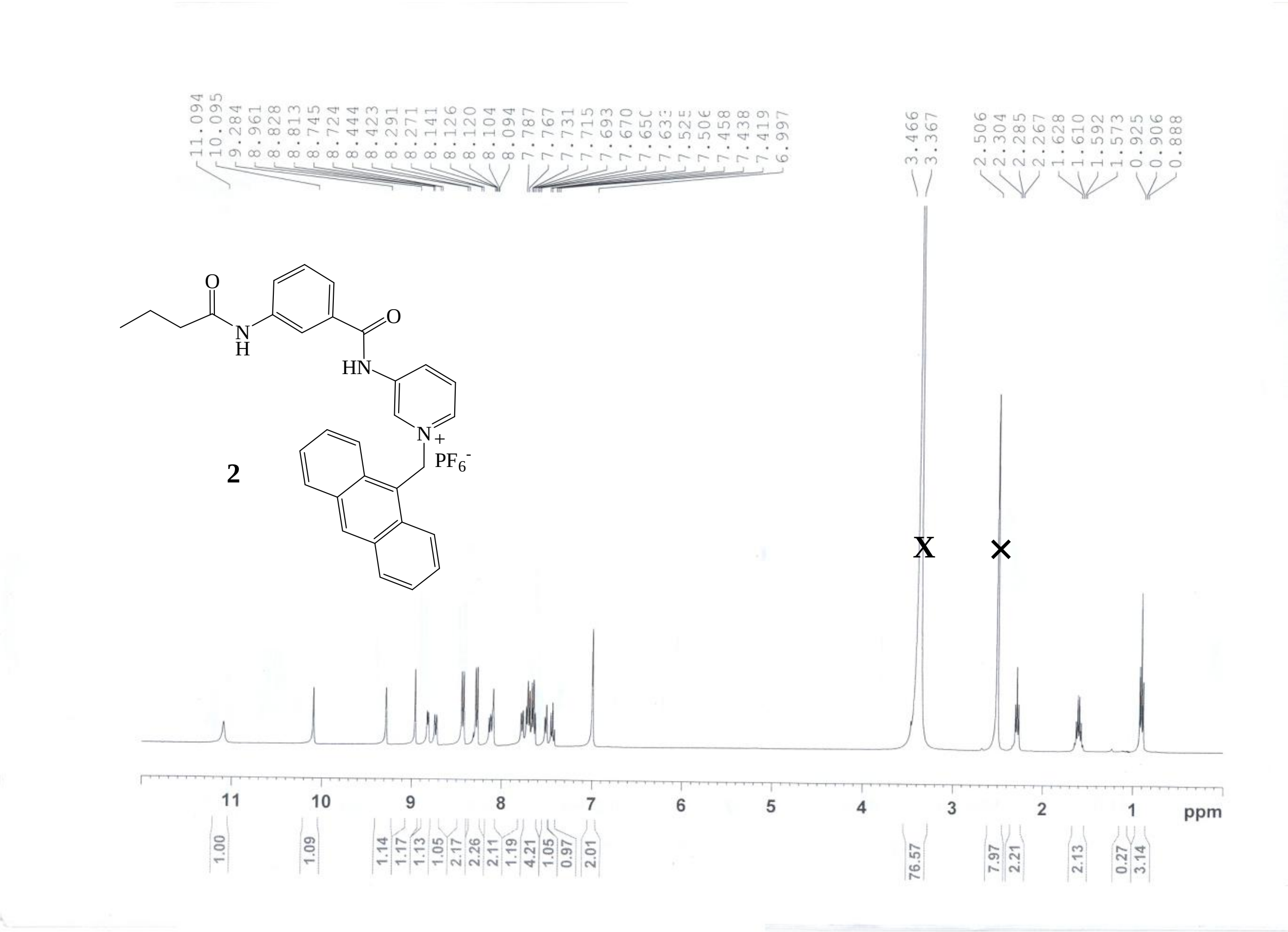

^{13}C NMR (100 MHz, *d_6*-DMSO)

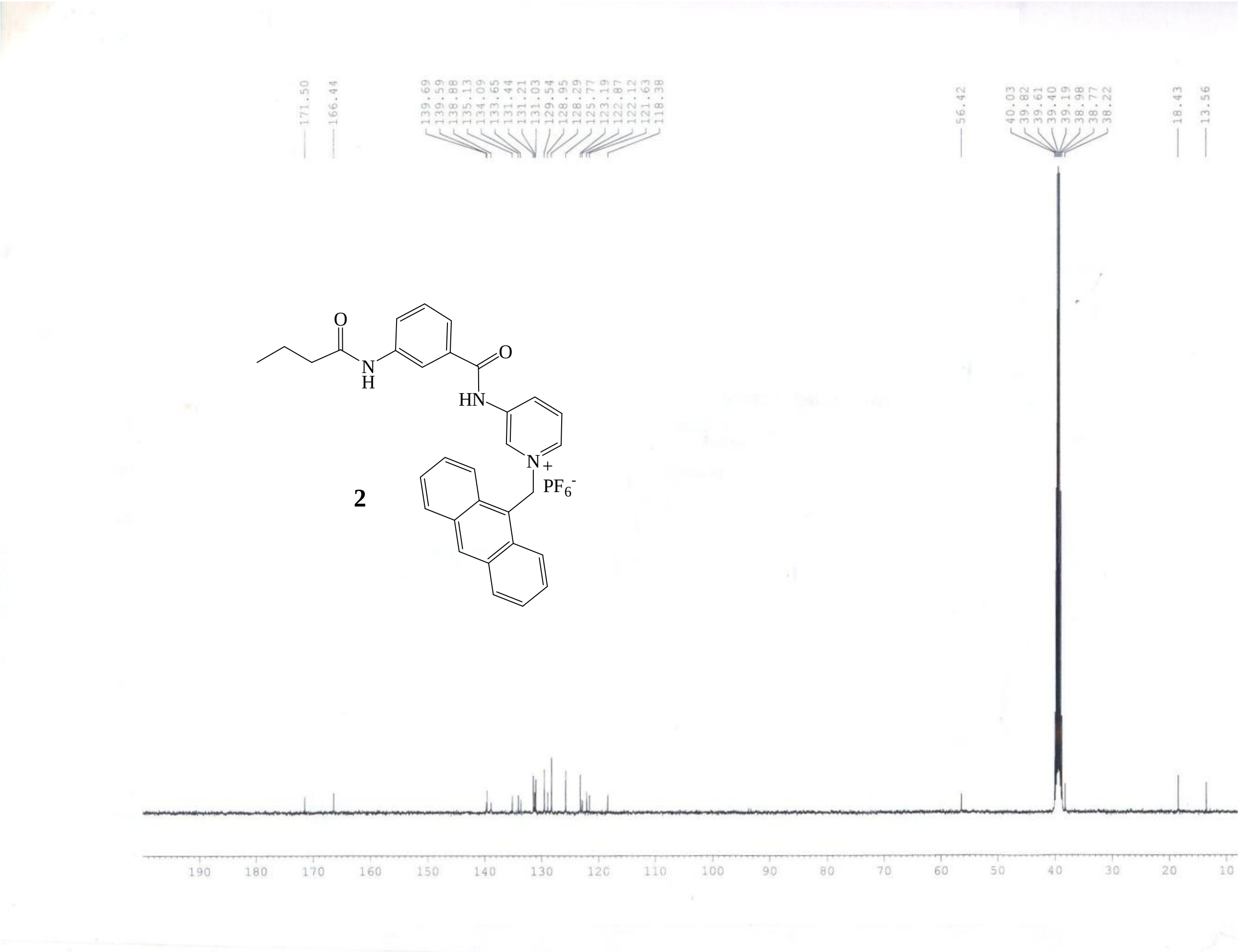

Supplement: File 1 — Detailed experimental data for 1 and 2. [file Beilstein_J_Org_Chem-06-1211-s001.pdf]
